# Azimuthal modulation of electromagnetically induced grating using structured light

**DOI:** 10.1038/s41598-021-00141-9

**Published:** 2021-10-20

**Authors:** Seyyed Hossein Asadpour, Teodora Kirova, Jing Qian, Hamid R. Hamedi, Gediminas Juzeliūnas, Emmanuel Paspalakis

**Affiliations:** 1 Department of Physics, Iran University of Science and Technology, Tehran, Iran; 2 Institute of Atomic Physics and Spectroscopy, University of Latvia, Riga, 1004 Latvia; 3 State Key Laboratory of Precision Spectroscopy, Quantum Institute for Light and Atoms, Department of Physics, School of Physics and Electronic Science, East China Normal University, Shanghai, 200062 China; 4 Institute of Theoretical Physics and Astronomy, Vilnius University, 10257 Vilnius, Lithuania; 5 Materials Science Department, School of Natural Sciences, University of Patras, 265 04 Patras, Greece

**Keywords:** Physics, Quantum physics, Theoretical physics

## Abstract

We propose a theoretical scheme for creating a two-dimensional Electromagnetically Induced Grating in a three-level $\Lambda$-type atomic system interacting with a weak probe field and two simultaneous position-dependent coupling fields—a two dimensional standing wave and an optical vortex beam. Upon derivation of the Maxwell wave equation, describing the dynamic response of the probe light in the atomic medium, we perform numerical calculations of the amplitude, phase modulations and Fraunhofer diffraction pattern of the probe field under different system parameters. We show that due to the azimuthal modulation of the Laguerre–Gaussian field, a two-dimensional asymmetric grating is observed, giving an increase of the zeroth and high orders of diffraction, thus transferring the probe energy to the high orders of direction. The asymmetry is especially seen in the case of combining a resonant probe with an off-resonant standing wave coupling and optical vortex fields. Unlike in previously reported asymmetric diffraction gratings for PT symmetric structures, the parity time symmetric structure is not necessary for the asymmetric diffraction grating presented here. The asymmetry is due to the constructive and destructive interference between the amplitude and phase modulations of the grating system, resulting in complete blocking of the diffracted photons at negative or positive angles, due to the coupling of the vortex beam. A detailed analysis of the probe field energy transfer to different orders of diffraction in the case of off-resonant standing wave coupling field proves the possibility of direct control over the performance of the grating.

## Introduction

When a strong coupling field in an Electromagnetically Induced Transparency (EIT)^1,2^ scheme is replaced by a standing-wave (SW), the so called Electromagnetically Induced Grating (EIG) is observed. In this case the traveling-wave (TW) probe field can be diffracted into higher order directions, due to the spatial periodic modulation for the absorption and dispersion of the medium, implemented by the SW field. EIG was first proposed by Ling et al.^3^, observed by Mitsunaga and Imoto in sodium atoms^4^, and later widely investigated in different systems^5–7^, including Rydberg atoms^8,9^. EIG has also been extended to two-dimensions in multi-level atomic systems^10^, involving non-linear modulation^11^, as well as Raman processes^12^.

EIGs may provide flexible amplitude and phase modulation of the weak probe beam, as well as manipulation of the intensity distribution of different orders of the grating. In the process of amplitude modulation the amplitude of the carrier wave is changed accordingly to the amplitude of the modulating signal, while the carrier phase and frequency remain constant. The carrier wave amplitude is modified in order to send data or information, usually over long distance. On the other hand, during phase modulation the phase of the carrier wave changes accordingly to the amplitude of the modulating signal, keeping the carrier amplitude and frequency constant. Phase modulation is used to transfer data/information for example in mobile systems. While phase modulation is similar to the process of frequency modulation, in phase modulation the frequency of the carrier signal is not increased.

It is well known, that in ordinary media the formed gratings typically diffract light symmetrically. In recent years it has been demonstrated, that in the PT-symmetric media it is possible to observe an asymmetric diffraction pattern since light undergoes spatially modulated refractive index^13^. The realization of one-and two-dimensional asymmetric gratings in optical PT-symmetric structures has drawn the attention of many research groups^14–25^.

Due to their adjustable optical properties EIGs have found many applications in all areas requiring diffraction gratings. Some examples include improving the structure of photonic band gaps by optically induced lattice^26^, storing propagated light through an atomic medium^27^ and construction of all-optical beam splitting and fanning^28^. Diffraction of light via EIGs can also be employed to form electromagnetically induced Talbot effect with applications in 2D ultra-cold atoms imaging^29^. Multi-component vector solitons based on EIG have been introduced^30^ and 2D surface solitons of four-wave mixing signals have been experimentally demonstrated in an optically induced atomic lattice^31^. Investigations of 2D and 3D EIGs also rise interest in terms of applications to spontaneously generated coherence^32^, surface plasmons in hybrid quantum dot-metal nanoparticles^33^, as well as atom localization in multi-level systems^34^.

A number of interesting effects also arise when atoms interact with the so called optical vortex beams, e.g. light beams carrying Orbital Angular Momentum (OAM). Such beams have a ring-shape intensity profile and a helical phase, which gives rise to a dark spot with no intensity in the center^35^. The OAM of light provides an additional degree of freedom in manipulation of optical information during the storage and retrieval of slow light, leading to novel applications in optical technologies such as data transmission, optical communication^36,37^, optical tweezers^38^, and quantum information^39^. The light-matter interaction using optical vortices results also in light-induced-torque^40^, second harmonic generation^41^, four-wave mixing^42^, spatially dependent EIT^43,44^, and vortex slow light^45,46^. Exchange of OAM modes in multi-level quantum systems has been extensively studied by the Juzeliūnas group^47–50^, while these schemes were recently extended also to applications in semiconductor quantum-dot molecules^51^ and semiconductor quantum wells^52,53^.

Optical vortices, however, have rarely been applied to atomic schemes in one- and two-dimensional EIG configurations. Since the OAM of light gives us additional degrees of freedom, we expect such an application to open additional possibilities for control over the EIG performance parameters. In the present work we study the effects of the simultaneous interaction of atomic three-level EIG scheme with a standing wave coupling field and a laser beam carrying OAM. The performed analytical and numerical studies show that the amplitude, phase modulations and Fraunhofer diffraction pattern of the probe field have quite different behaviours depending on whether the applied probe, standing wave and optical vortex fields are on- or off-resonant. In addition, by changing the azimuthal index of the optical vortex field, the thus created two-dimensional EIG acquires an asymmetric character, transferring the probe field energy to the high orders of direction. The behavior of the different orders of diffraction in the case of off-resonant standing wave and optical vortex coupling fields for different values of the OAM number gives us a better understanding of the possibilities to achieve a good control of the EIG. It is worth noting, that while previously the asymmetric diffraction grating has been reported for PT-symmetric structures, in our work we show that for the asymmetric diffraction grating the parity time symmetric structure is not necessary. The possibility to achieve asymmetric gratings without the presence of PT-symmetric structure is outlined in Ref.^54^, and in some specific cases in Ref.^24^. The type of asymmetric diffraction in our studies can be better explained by considering the constructive and destructive interference effect between the amplitude and phase modulations of the grating system, leading to diffracted photons only at positive or negative angles. Due to the action of the LG beam there exists a complete blocking of the diffracted photons at negative or positive angles for different number of the OAM. The EIG is controlled easily only by changing the vorticity of the Laguerre–Gaussian coupling field, and its experimental realization should be similar to the typical atomic EIG experiments^4,5^. We expect possible applications of the proposed EIG in adding flexibility to different quantum devices, e.g. all-optical quantum switches, logic gates, or other EIT-based devices, making our findings relevant for all-optical information processing and atom-manipulation.

The paper is organized as follows. In “Introduction” we introduce the excitation scheme and the theoretical model we use, and present the basic set of equations by solving analytically the coupled Maxwell–Bloch equations. The numerical results and their discussion are presented in “Theoretical model and equations”, followed by a short discussion on possible experimental applications. The concluding Section summarizes our findings.

## Theoretical model and equations

**Figure 1:**
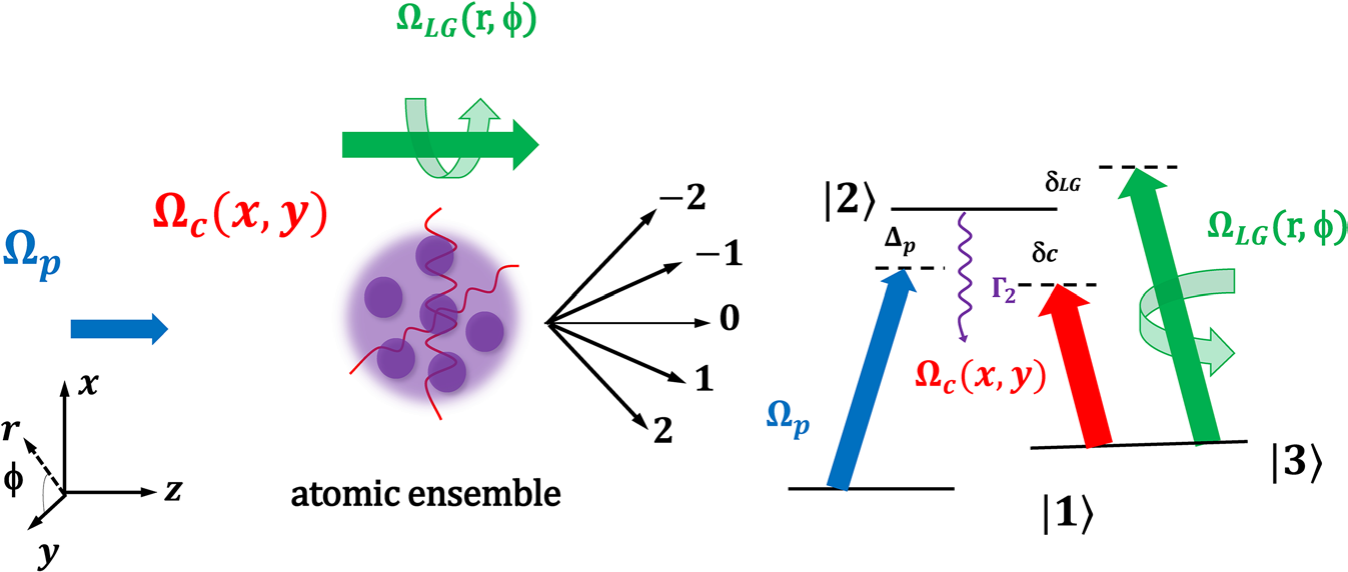
(**a**) Schematic representation of the EIG scheme. Atomic ensemble consisting of $\Lambda$-level atoms is excited by a weak TW probe field and simultaneously coupled by strong SW and LG fields. Due to the phase and amplitude modulations exerted by the coupling fields, the incident probe field is diffracted into high-order diffraction after propagating through such an ensemble. (**b**) Atomic energy levels scheme. Other details are given in the text. The figure was prepared using Microsoft PowerPoint, Microsoft Office Professional Plus 2016, www.microsoft.com.

We consider a three-level atomic system arranged in a $\Lambda -$ configuration of atomic levels involving two ground states $|1\rangle$ and $|3\rangle$, and an excited state $|2\rangle$, as shown in Fig. 1 A weak probe field in the form of a travelling wave induces the atomic transition $|1\rangle \rightarrow |2\rangle$, while the transition $|2\rangle \rightarrow |3\rangle$ is driven simultaneously by a coupling field in the form of a standing wave (SW) along *x*, *y*- direction and a vortex Laguerre–Gaussian (LG) field.

The probe field can be expressed as:

1 $$\begin{aligned} E_p(x,z,t)=\frac{1}{2}E_{p}e^{-i\omega _{p}t+ik_{p}z}+c.c., \end{aligned}$$

where $E_{p}$ is the amplitude of the field and *c*.*c*. stands for the complex conjugate operation, while the SW coupling field along the x, y-direction has the form:

2 $$\begin{aligned} E_c(x,y,t)=\frac{1}{2}E_{c}[\sin (\pi x/\Lambda _{x})+\sin (\pi y/\Lambda _{y})]e^{-i\omega _{c}t}+c.c. \end{aligned}$$

Here $\Lambda _{x}=\pi /k_{cx}$ and $\Lambda _{y}=\pi /k_{cy}$ describe the spatial periodicity of the standing waves.

The Rabi frequencies of the probe and two coupling fields are then given by:

3 $$\begin{aligned}&\Omega _p=\vec{\mu _{12}}\cdot \vec {E_{p}}/2\hbar , \end{aligned}$$

4 $$\Omega _c=\Omega _{c0}[\sin (\pi x/\Lambda _{x})+\sin (\pi y/\Lambda _{y})], $$

5 $$\begin{aligned}&\Omega _{LG}=\Omega e^{-\frac{r^2}{\omega ^2}} \left( \frac{r}{\omega } \right) ^{ |l |} e^{il\phi }, \end{aligned}$$

where $r=\sqrt{x^2+y^2}$ is the radial distance from the axis of the LG beam, $\phi$ denotes the azimuthal angle, *l* is an integer representing the vorticity of the LG beam and $\omega$ stands for the beam waist parameter. The parameters $\Omega _{c0}=\mathbf {\mu _{23}}\cdot \mathbf {E_{c}}/2\hbar$ and $\Omega =\mu _{23} E_{LG}/2\hbar$ are the initial peak amplitudes of the standing wave and the LG coupling fields, respectively.

Under the electric dipole approximation and Rotating-Wave Approximation (RWA)^55^, the Hamiltonian of the system is given by in the interaction representation, following^56^:

6 $$\begin{aligned} H_{int}=-\hbar \Delta _p | 2 \rangle \langle 2 | -\hbar (\Delta _{p}-\Delta _{c}) |3\rangle \langle 3 | -\hbar [\Omega _{p}|2\rangle \langle 1| +(\Omega _{c}+\Omega _{LG})e^{-i\delta t}|2\rangle \langle 3 | +h.c.]. \end{aligned}$$

Here $\Delta _{p}=\omega _{p}-\omega _{31}$ denotes the detuning of the probe laser field. We have introduced the additional notation $\Delta _{c}=\omega _{32}-(\delta _{c}+\delta _{LG})/2$, whrere $\delta _{c}$ and $\delta _{LG}$ represent the detunings of the SW and LG lasers. For the purposes of creating the EIG we need to have equal detunings of the SW and LG fields at all times, e.g. $\delta _{c}=\delta _{LG}=\delta$. On the other hand, $\omega _{ij}, (i, j=1...3)$ is the resonant frequency of the atomic transition $|i\rangle \rightarrow |j\rangle$, and $\omega _{p}$ stands for the probe laser frequency.

The behaviour of the system is governed by the density-matrix equation of motion, which in the interaction picture has the form:

7 $$\begin{aligned} \frac{\partial \rho }{\partial t}=-\frac{i}{\hbar }[H_{int},\rho ]+{\fancyscript{L}} \rho , \end{aligned}$$

where $H_{int}$ is the interaction Hamiltonian, $\rho$ stands for the density-matrix operator, and the last term ${\fancyscript{L}}\rho =-\{\Gamma , \rho \}/2= -(\Gamma \rho +\rho \Gamma )/2$ describes the effects of different decay mechanisms in the system due to spontaneous emission of the atomic levels, dephasing terms, etc.

From Eq. (7) the equations of motion for the density matrix elements become, following^3^:

8 $$\begin{aligned} \frac{\partial \rho _{11}}{\partial t}= & {} \Gamma _{21} \rho _{22} +\Gamma _{31} \rho _{33}+ i\Omega _{p}^{*}\rho _{21}-i\Omega _{p}\rho _{21}, \nonumber \\ \frac{\partial \rho _{33}}{\partial t}= & {} \Gamma _{23} \rho _{22} -\Gamma _{31} \rho _{33}+ i\left( \Omega _{c}+\Omega _{LG}\right) e^{-i\delta t}\left( \rho _{23}-\rho _{32}\right) , \nonumber \\ \frac{\partial \rho _{21}}{\partial t}= & {} \left( -\gamma _{21} +i\Delta _{p}\right) \rho _{21}+i\Omega _{p}\left( \rho _{11}-\rho _{22}\right) +i\left( \Omega _{c}+\Omega _{LG}\right) e^{i\delta t}\rho _{31}, \nonumber \\ \frac{\partial \rho _{23}}{\partial t}= & {} \left( -\gamma _{23} +i\Delta _{p}\right) \rho _{23}+i\Omega _{p}\rho _{13} +i\left( \Omega _{c}+\Omega _{LG}\right) ^{i\delta t}\left( \rho _{33}-\rho _{22}\right) , \nonumber \\ \frac{\partial \rho _{31}}{\partial t}= & {} \left[ -\gamma _{31} +i \left( \Delta _{p}-\Delta _{c}\right) \right] \rho _{31} +i\left( \Omega _{c}+\Omega _{LG}\right) e^{-i\delta t}\rho _{21}-i\Omega _{p}\rho _{32} \end{aligned}$$

where $\Gamma _{ij}$ and $\gamma _{ij}$ are the population decay and the dephasing rate between levels *i* and *j*, respectively. For simplicity, we assume $\Gamma _{2}=\Gamma _{21}+\Gamma _{23}=\gamma$ and all the other parameters take $\gamma$ as a unit. In the above we also use the Hermitian condition for the density matrix $\rho _{ij}=\rho _{ji}^{*}, i\ne j$, as well as the condition for total population conservation $\rho _{11}+\rho _{22}+\rho _{33}=1$, as the atomic system is assumed to be closed.

Solving the equations of motions under the assumption of a weak probe field, we can obtain an expression for the matrix element $\rho _{12}$ which holds to all orders of the coupling fields, but depends linearly on the weak probe field. The real and imaginary part of $\rho _{12}$ are given as follows, similarly to^3^:

9 $$\begin{aligned}&Im(\rho _{12})= \frac{[\gamma _{31}^2+\Delta ^2]+\gamma _{13}|\Omega _{c}+\Omega _{LG}|^2}{(1+\Delta _{p}^2)[\gamma _{31}^2+\Delta ^2]+2[\gamma _{31}-\Delta _{p}\Delta ]|\Omega _{c}+\Omega _{LG}|^2+|\Omega _{c}+\Omega _{LG}|^4}, \end{aligned}$$

10 $$\begin{aligned}&Re(\rho _{12})= \frac{-\Delta _{p}[\gamma _{31}^2+\Delta ^2]+\Delta |\Omega _{c}+\Omega _{LG}|^2}{(1+\Delta _{p}^2)[\gamma _{31}^2+\Delta ^2]+2[\gamma _{31}-\Delta _{p}\Delta ]|\Omega _{c}+\Omega _{LG}|^2+|\Omega _{c}+\Omega _{LG}|^4}, \end{aligned}$$

where we have introduced the notation $\Delta =\Delta _{p}-\Delta _{c}$.

In our analytical expressions the dephasing rate between levels $|3\rangle$ and $|1\rangle$ $\gamma _{31}$ is included, while the term $\gamma _{21}$, although considered in our model [see Eqs. (8)], is omitted due to some simplifications. In the above equations one can see clearly the effect of the term $|\Omega _c+\Omega _{LG}|^2$, representing the simultaneous action of both the *SW* and *LG* fields in the system. Rewriting the form of the LG field in Eq. (5) as $\Omega _{LG}=\Omega ^{'} e^{il\phi }$, we can expand $|\Omega _c+\Omega _{LG}|^2=|\Omega _c+\Omega ^{'} e^{il\phi }|^2=\Omega _{c}^{2}+2\Omega _{c}\Omega ^{'}\cos (l\phi )+\Omega ^{'2}$, so we obtain a direct dependence on *l*. Thus we can conclude that the azimuthal angle of the vortex field plays a crucial role in Eqs. (9) and (10) and the diffraction pattern of the probe light can be controlled by changing the OAM number of the vortex light, leading to the possibility for adjusting the EIG pattern on demand.

It is worth noting, that the simultaneous coupling by the $\Omega _s$ and $\Omega _{LG}$ fields is exactly the one giving the opportunity for such a control. For comparison, if the levels $|2\rangle$ and $|3\rangle$ were interacting with the standing field only, while another atomic transition was coupled by the vortex field, the parameters $|\Omega _{c}|^2$ and $|\Omega _{LG}|^2$ would appear in the equations separately and the results would be independent of the azimuthal angle of the vortex field. Thus, in our scheme we take advantage of the additional degrees of freedom provided by the OAM of the LG light, in order to gain an extra control over the EIG performance parameters, going beyond the atomic EIG schemes previously studied in the literature.

The dynamic response of the probe light in the atomic medium is described by Maxwell’s wave equation. When the slowly varying envelope approximation and the steady state regime are considered, it can be concluded that:

11 $$\begin{aligned} \frac{\partial E_{p}}{\partial z}=\frac{ik_{p}}{2\epsilon _{0}}P(\omega _{p}) \quad \text {or} \quad \frac{\partial E_{p}}{\partial z^{'}}=[-\mathrm {Im}(\chi )+i\mathrm {Re}(\chi )]E_{p}. \end{aligned}$$

In the above $z^{'}=(N\mu _{21}^{2}/2\hbar \epsilon _{0})k_{p}z$ is treated as the unit for *z*, with $k_{p}=2\pi /\lambda$ and $\chi =\gamma _{31}\rho _{12}$ represents the optical susceptibility of the medium. The normalized transmission function for the interaction length *L* of the atomic sample is then given by:

12 $$\begin{aligned} T(x,y)=e^{-Im(\chi )L}e^{{iRe(\chi )L}}, \end{aligned}$$

where the first (second) term in the exponential corresponds to the grating amplitude (phase) modulation, respectively. The transmission function *T*(*x*, *y*) depends on the localization of the atom and the EIT.

The Fraunhofer (far-field diffraction) equation can be obtained by the Fourier transformation of *T*(*x*, *y*) as:

13 $$\begin{aligned} I_{p}(\theta _{x},\theta _{y})=|E(\theta _{x},\theta _{y})|^{2}\frac{\sin ^{2}(M\pi R_{x} sin\theta _{x})}{M^{2}\sin ^{2}(\pi R_{x} \sin \theta _{x})}\frac{\sin ^{2}(N\pi R_{y} \sin \theta _{y})}{N^{2}\sin ^{2}(\pi R_{y}\sin \theta _{y})}, \end{aligned}$$

where

14 $$\begin{aligned} E(\theta _{x},\theta _{y})=\int _{0}^{1} T(x,y)\exp (-i2\pi x \sin \theta _{x})\exp (-i2\pi y \sin \theta _{y}){dxdy} \end{aligned}$$

is the Fraunhofer diffraction of a single space period. Here $\theta _{x}$ and $\theta _{y}$ stand for the diffraction angles with respect to the *z* direction, $R_{x}=\Lambda _{x}/\Lambda$, $R_{y}=\Lambda _{y}/\Lambda$, and *M*(*N*) is the number of spatial periods of the grating irradiated by the probe beam. For simplicity we assume equal values of $R_{x}$ and $R_{y}$, e.g. $R_{x}=R_{y}=R$. The diffraction orders *m*, *n* are determined by the grating equations $\sin \theta _{x}=m/x$ and $\sin \theta _{y}=n/y$. Eqs. (11)–(14) represent the typical grating equations found in most EIG-related works, however we include them here for the sake of the completeness.

## Numerical results

In this section we present our main results and discuss the effects of the *LG* field azimuthal parameter *l* on the EIG, as well as analyze the controllability of the diffraction efficiency of the grating via tuning of different parameters. Depending on the appropriate choice of *l* and the probe and coupling fields detunings, the probe light can be diffracted into four regions, i.e. region *I* $(0\le \sin {\theta _{x}}\le 1$, $0\le \sin {\theta _{y}}\le 1 )$, region *II* $(0\le \sin {\theta _{x}}\le 1$, $-1\le \sin {\theta _{y}}\le 0 )$, region *III* $(-1\le \sin {\theta _{x}}\le 0$ $0\le \sin {\theta _{y}}\le 1 )$, and region *IV* $(-1\le \sin {\theta _{x}}\le 0$, $-1\le \sin {\theta _{y}}\le 0 )$. Here we note, that in the two-photon resonance case, the probe energy remains in the zero-order of diffraction. As in this work we are interested in transferring the probe energy from zero-order to high order of diffraction, we will not discuss this case in detail.

### Off-resonant probe and resonant coupling fields

First, we consider the condition where the SW and LG coupling lasers are in resonance with the transition $|2\rangle \rightarrow |3\rangle$, e.g. $\delta =0$, while the weak probe is tuned far from the transition $|2\rangle \rightarrow |1\rangle$. From Eqs. (9) and (10) one can find that for the case $\Delta _{p}\ne 0$ and $\Delta _{c}=0$ we expect the real and imaginary part of the susceptibility to become non-zero. Therefore, some of the probe energy may transfer from zero order to high orders of diffraction when we change the OAM number of the vortex field.

**Figure 2:**
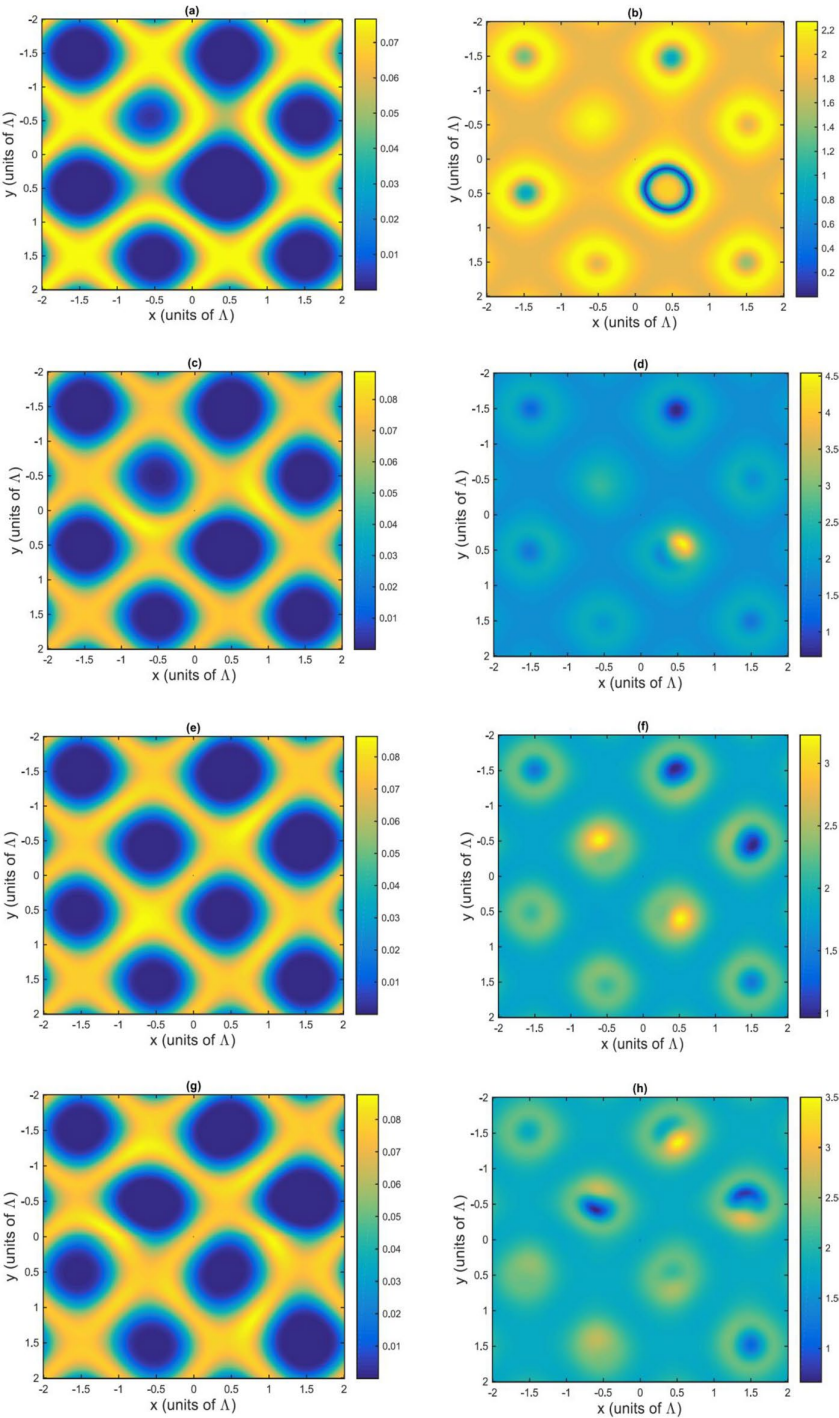
Amplitude (**a**,**c**,**e**,**g**) and phase (**b**,**d**,**f**,**h**) modulations in the case of off-resonant probe and resonant SW and LG fields. The selected parameters are $\gamma _{31}=0.1\gamma$, $\Delta _{p}=2.2\gamma$, $\delta =0$, ${\Omega =\Omega _{c0}}=\gamma$, $\omega =\gamma$, $M=N=5$, $L=25$, and $R=4$. (**a**,**b**) $l=0$; (**c**,**d**) $l=1$; (**e**,**f**) $l=2$; (**g**,**h**) $l=3$.

To check these predictions, in Fig. 2 we display the amplitude (a, c, e, g) and phase (b, d, f, h) modulations when $\Delta _{p}=2.2\gamma$ for different values of the azimuthal index parameter $l=0, 1, 2, 3$.

For $l=0$ a phase modulation accompanied by an absorption modulation is obtained, the propagation of the probe beam being mainly affected by the phase modulation. As the azimuthal index of the LG beam is increased to $l=1$ (Fig. 2c,d), the amplitude and phase modulation are enhanced. Further increasing to $l=2, 3$ has no effect on the behavior of the amplitude modulation, as shown in Fig. 2e,g. In contrast, the phase modulations in Fig. 2f,h are significantly changed, e.g. their value reduces respectively.

**Figure 3:**
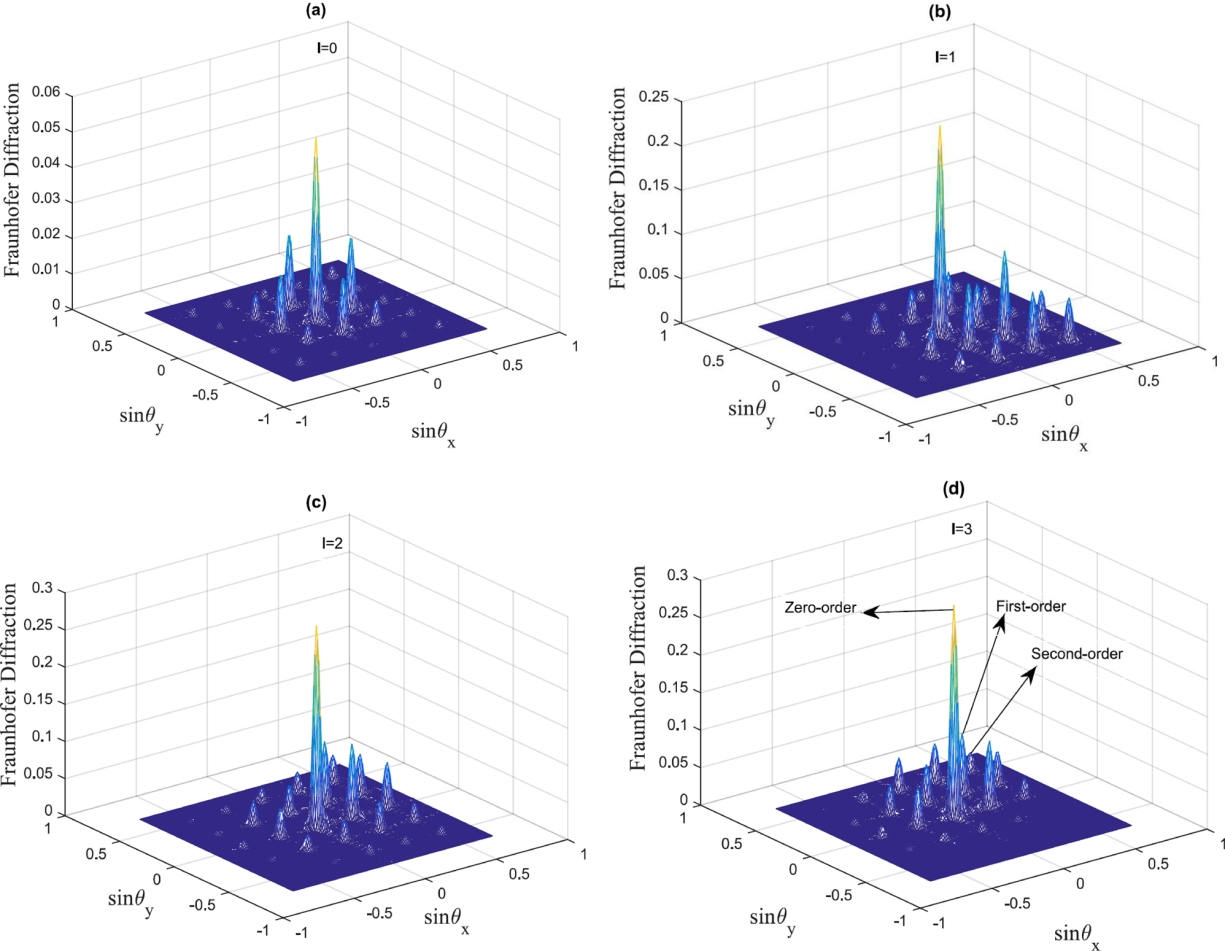
Fraunhofer diffraction pattern in the case of off-resonant probe and resonant SW and LG fields. The selected parameters are $\gamma _{31}=0.1\gamma$, $\Delta _{p}=2.2\gamma$, $\delta =0$, ${\Omega =\Omega _{c0}}=\gamma$, $\omega =\gamma$, $M=N=5$, $L=25$, $R=4$. (**a**) $l=0$; (**b**) $l=1$; (**c**) $l=2$; (**d**) $l=3$.

In Fig. 3 we plot the form of the Fraunhofer diffraction patterns corresponding to the off-resonant probe field $\Delta _{p}=2.2\gamma$ when the azimuthal index *l* is varied from 0 to 3.

The diffraction pattern in Fig. 3a illustrates that the phase modulation deflects a significant portion of the probe energy into the first-order of direction intensities. When $l=1$ (Fig. 3b) the zeroth and high orders of diffraction increase and more of the probe energy transfers to the high orders of direction. Interestingly, an asymmetric grating is observed here, due to the azimuthal modulation of the *LG* field. The Fraunhofer diffraction peaks shift their positions via varying of *l* to 2, 3, as seen in Fig. 3c,d.

Analyzing Figs. 2 and 3 we can conclude that for the case $l=0$, a symmetric EIG pattern is obtained. At the same time, when $l=1$, the amplitude and phase modulation of the EIG induce constructive interference in region *II*, while they induce destructive interference in regions *I*, *III* and *IV*. The constructive interference in region *II* leads to build up of the probe intensity, while the destructive interference reduces the intensity of the probe field in regions *I*, *III* and *IV*. However, by changing the azimuthal index of the of the vortex field to $l=2$ and 3, the diffraction pattern of the probe beam can be shifted from region *II* to region *I*, respectively. In addition to the above, the diffraction pattern of the probe light can be switched from one region to another only by modulation of the azimuthal index of the vortex field.

The main result in this subsection is related to changing the positions of the high order of diffraction due to altering the OAM number of the vortex field. We observe a symmetric pattern for the amplitude grating and a non-symmetric pattern for the phase grating when the *OAM* number of the vortex field is altered. Most of the probe energy, however, remains in the zero order of diffraction due to the symmetric pattern of the amplitude grating and some portion of energies transfer to high order of diffraction, leading to an asymmetric grating with small efficiency.

### Resonant probe and off-resonant coupling fields

To increase the efficiency of the high-order diffraction, we must move into a parameter regime where the phase modulation is significant. Ideally, we wish to create a medium that is completely transparent to the probe light, but has a phase modulation across the probe beam. Therefore, in this subsection we will work in a regime where the SW and LG coupling fields are off-resonant from the transition $|2\rangle \rightarrow |3\rangle$, while the weak probe is tuned to transition $|1\rangle \rightarrow |2\rangle$. Due to this condition, as seen from Eqs. (9) and (10), a good level of transparency and a large phase modulation should be possible in the system. In this case we expect that most of the probe energy transfers from zero to high orders of diffraction in an asymmetric pattern when the OAM number of the vortex field is changed, thus creating an EIG with good efficiency.

**Figure 4:**
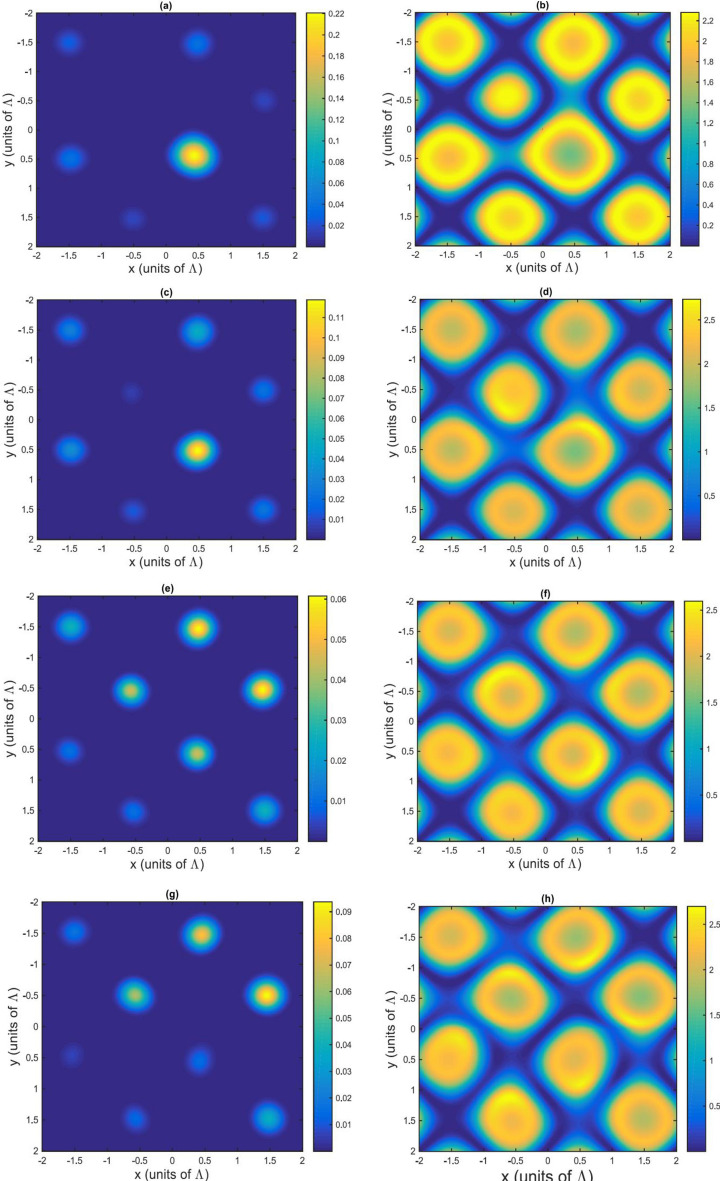
Amplitude (**a**,**c**,**e**,**g**) and phase (**b**,**d**,**f**,**h**) modulations in the case of resonant probe and off-resonant SW and LG fields. The selected parameters are $\gamma _{31}=0.1\gamma$, $\Delta _{p}=0$, $\delta =2.2\gamma$, ${\Omega =\Omega _{c0}}=\gamma$, $\omega =\gamma$, $M=N=5$, $L=25$, $R=4$. (**a**,**b**) $l=0$; (**c**,**d**) $l=1$; (**e**,**f**) $l=2$; (**g**,**h**) $l=3$.

By analyzing the numerical results in Fig. 4, we indeed find that a non-symmetric pattern for the amplitude (a, c, e, g) and a symmetric pattern for the phase (b, d, f, h) modulations are achieved for this condition of parametric medium.

**Figure 5:**
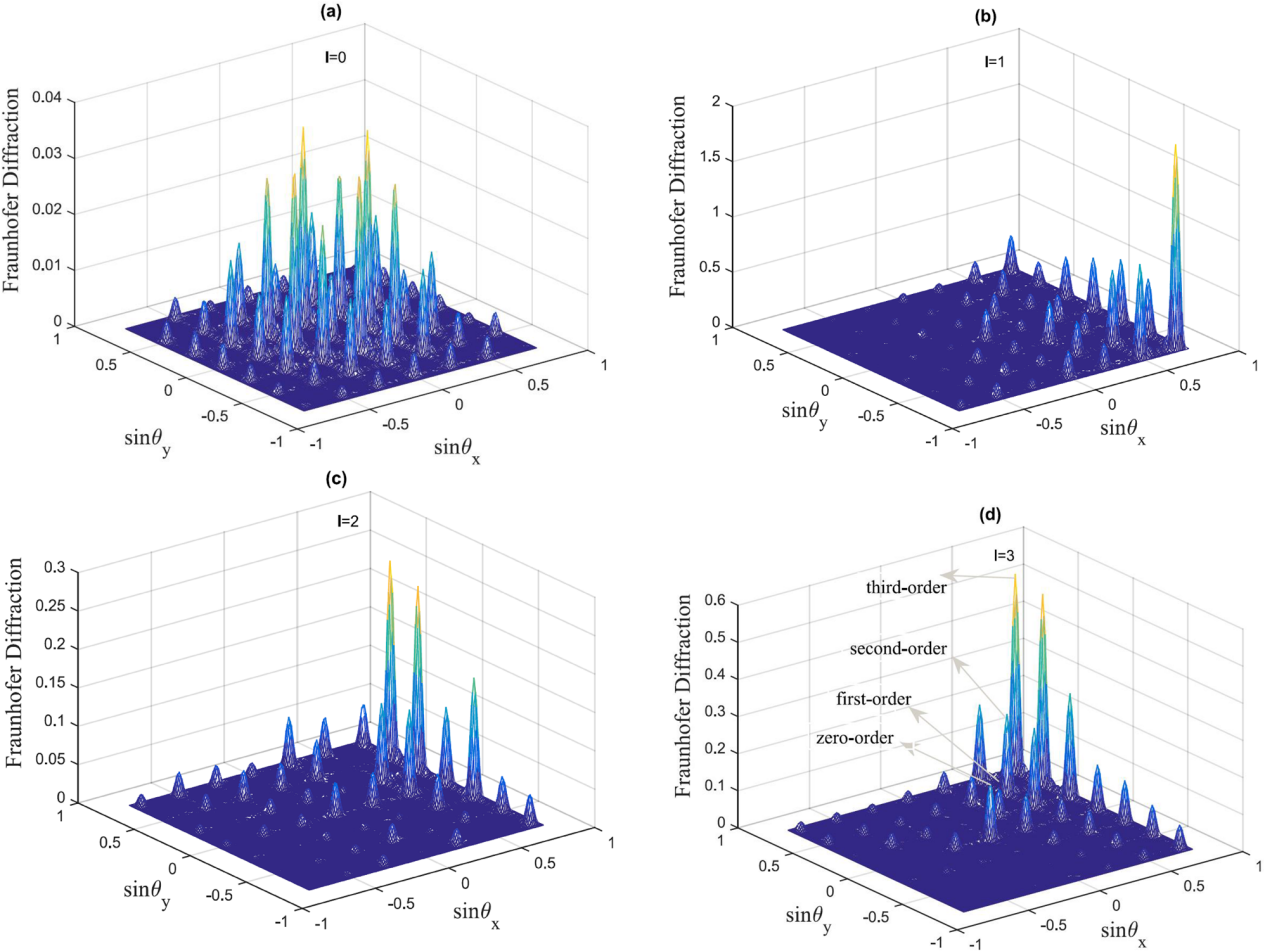
Fraunhofer diffraction pattern in the case of resonant probe and off-resonant SW and LG fields. The selected parameters are $\gamma _{31}=0.1\gamma$, $\Delta _{p}=0$, $\delta =2.2\gamma$, ${\Omega =\Omega _{c0}}=\gamma$; $\omega =\gamma$, $M=N=5$, $L=25$, $R=4$. (**a**) $l=0$; (**b**) $l=1$; (**c**) $l=2$; (**d**) $l=3$.

The value of the amplitude modulation (Fig. 4a,c,e,g) in x, y plane is almost zero and is non-zero only in few points. At the same time, the phase modulation (Fig. 4b,d,f,h) has symmetric behavior and its value becomes significant.

Studying the behavior of the Fraunhofer diffraction, as shown in Fig. 5a–d, we observe that the position of the asymmetric diffraction patterns may also shift when we alter the OAM number from $l=0$ (shown in Fig. 5a) to $l=3$ (Fig. 5d). This type of asymmetry can be explained by the effect of interference between the asymmetric modulation of the amplitude grating and the symmetric modulation of the phase grating.

Looking into the energy transfer, in the case of $l=0$ (Fig. 5a) a symmetric pattern for the Fraunhofer diffraction is obtained and most of the probe energy is transferred to the high orders of diffraction due to phase modulation of EIG. However, when the OAM number is $l=1$, most of the probe light energy moves to region *II* and mostly gathers near $\sin {\theta _{x}}=0.75$ and $\sin {\theta _{y}}=-0.75$. By changing the OAM number to $l=2$, the diffracted light is switched to region *I* and most of the probe light energy gathers near $\sin {\theta _{x}}=0.75$ and $\sin {\theta _{x}}=-0.5$. Finally, for $l=3$, the diffracted light also remains in region *I*, but most of the probe energy transfers to $\sin {\theta _{x}}=0.75$ and $\sin {\theta _{y}}=-0.75$, respectively.

It is worth noting, that our calculations are performed for equal values of *M* and *N* (*M* and *N* are the numbers of the spatial periods along the *x* and *y* axes of the grating illuminated by the probe beam). The case $M\ne N$ may lead to changing the number of the diffracted orders in the *x* and *y* axes, which results in the probe energy gathering mostly in the *x* or *y*-axes.

We can conclude that due to the off-resonance of the two coupling lasers and the resonance of the probe light, more energy is transferred to high order maxima and the region of the diffracted light is switched by changing the vortex light OAM number. Therefore, the intensity distribution of the diffraction can be controlled by adjusting the probe and coupling detunings and OAM number of the LG light. The latter gives an advantage of our EIG configuration, where simultaneously a SW and a LG coupling beams are applied, over other typical atomic 2D grating schemes in the literature.

### Experimental feasibility

The experimental evolution of diffraction pattern of probe light has been studied in different atomic or solid state medium^57–59^. For example, in three-level ladder type atomic system^58^, Yuan et al., have observed experimentally the diffraction pattern of the weak probe light in two dimensional optical atomic lattice. They have used two orthogonal standing wave lights which can be obtained via interference of two pairs of coupling laser fields. When the probe light is launched into it, a spatially modulated discrete diffraction pattern can be obtained at the output plane of the vapor cell under the EIT condition. In another study^57^, the diffraction patterns of the probe light in a coherent rubidium cascade system have been studied experimentally. They authors found that by adjusting the vapor temperature, laser detuning and intensity of the probe light the diffraction pattern can be controlled. Based on the above discussion, we believe that our proposed model may be interesting for experimental realization by considering the fact that the controlling of the diffraction pattern via vortex light has not yet been reported theoretically or experimentally in real atomic systems. Addressing the experimental feasibility of the proposed EIG scheme, the atomic $\Lambda$-configuration can be realized by using, for example, the Zeeman sublevels of $^{87}{Rb}$ cold atoms. The ground levels $|1>$ and $|3>$ can correspond to the sublevels $|F=1, m=0>$ and $|F=2, m=1>$ of the $5S_{1/2}$ state, while the $|F'=1, m'=0>$ sublevel of the $5P_{3/2}$ state can serve as the excited level $|2>$. The SW field can be produced via confinement in optical cavity/ Fabry–Perot resonator, or by using two counter-propagating laser beams. Having in mind the above considerations, the experimental set-up for observing the proposed 2D asymmetric grating can be similar to the ones found in the literature, for example^4,5^.

## Conclusion

In this work we have studied analytically and numerically the effects of the simultaneous coupling of an atomic three-level $\Lambda$-type scheme by a standing wave field along the *x*, *y*-direction and a laser beam carrying OAM in a EIG configuration. An ideal grating would achieve a complete transparency to the probe light with a phase modulation across the probe beam. Although it is impossible to realize such an ideal grating with our model, we show that by an azimuthal modulation of the Laguerre–Gaussian field (e.g. changing its vorticity) we can create a two-dimensional asymmetric grating, effectively transferring the probe field energy to the high orders of direction. The asymmetric effect is especially profound when a resonant probe and off-resonant standing wave and optical vortex coupling fields are applied. By analyzing the behavior of the different orders of diffraction in the case of off-resonant standing wave and optical vortex coupling fields for different values of the azimuthal number of the Laguerre–Gaussian beam, we explore the possibilities for direct control of the proposed EIG. Compared to other schemes, this grating is easily controlled by only adjusting the OAM number of the Laguerre–Gaussian coupling field. Thus, our scheme takes advantage of the additional degrees of freedom provided by optical vortices to gain additional control over the grating performance, going beyond configurations previously studied in the literature. Another advantage of our scheme is that a parity time symmetric structure is not necessary for achieving the asymmetry of the grating, unlike in previously reported asymmetric diffraction gratings for PT symmetric structures. By controlling the OAM number of the vortex field we can achieve a complete blocking of the diffracted photons at negative or positive angles, due to the interference effects between the amplitude and phase modulations of the system. Our findings are relevant for designing of new quantum devices such as all-optical quantum switches and logic gates, as well as improving the performance of other EIT-based devices. This may find applications in all-optical information processing and atom-manipulation technologies. The experimental confirmation of the asymmetrical two-dimensional grating proposed here should be easily realized similarly to other EIG experiments in atomic media.
